# Developmental Neurotoxic Effects of Percutaneous Drug Delivery: Behavior and Neurochemical Studies in C57BL/6 Mice

**DOI:** 10.1371/journal.pone.0162570

**Published:** 2016-09-08

**Authors:** Huali Wu, Junyi Feng, Wenting Lv, Qiaoling Huang, Mengsi Fu, Minxuan Cai, Qiangqiang He, Jing Shang

**Affiliations:** 1 State Key Laboratory of Natural Medicines, China Pharmaceutical University, Nanjing, China; 2 Jiangsu Key Laboratory of TCM Evaluation and Translational Research, China Pharmaceutical University, Nanjing, China; 3 Qinghai Key Laboratory of Tibetan Medicine Pharmacology and Safety Evaluation, Northwest Institute of Plateau Biology, Chinese Academy of Sciences, Xining, QingHai Province, PR China; University of Alabama at Birmingham, UNITED STATES

## Abstract

Dermatosis often as a chronic disease requires effective long-term treatment; a comprehensive evaluation of mental health of dermatology drug does not receive enough attention. An interaction between dermatology and psychiatry has been increasingly described. Substantial evidence has accumulated that psychological stress can be associated with pigmentation, endocrine and immune systems in skin to create the optimal responses against pathogens and other physicochemical stressors to maintain or restore internal homeostasis. Additionally, given the common ectodermal origin shared by the brain and skin, we are interested in assessing how disruption of skin systems (pigmentary, endocrine and immune systems) may play a key role in brain functions. Thus, we selected three drugs (hydroquinone, isotretinoin, tacrolimus) with percutaneous excessive delivery to respectively intervene in these systems and then evaluate the potential neurotoxic effects. Firstly, C57BL/6 mice were administrated a dermal dose of hydroquinone cream, isotretinoin gel or tacrolimus ointment (2%, 0.05%, 0.1%, respectively, 5 times of the clinical dose). Behavioral testing was performed and levels of proteins were measured in the hippocampus. It was found that mice treated with isotretinoin or tacrolimus, presented a lower activity in open-field test and obvious depressive-like behavior in tail suspension test. Besides, they damaged cytoarchitecture, reduced the level of 5-HT-5-HT1A/1B system and increased the expression of apoptosis-related proteins in the hippocampus. To enable sensitive monitoring the dose-response characteristics of the consecutive neurobehavioral disorders, mice received gradient concentrations of hydroquinone (2%, 4%, 6%). Subsequently, hydroquinone induced behavioral disorders and hippocampal dysfunction in a dose-dependent response. When doses were high as 6% which was 3 times higher than 2% dose, then 100% of mice exhibited depressive-like behavior. Certainly, 6% hydroquinone exposure elicited the most serious impairment of hippocampal structure and survival. The fact that higher doses of hydroquinone are associated with a greater risk of depression is further indication that hydroquinone is responsible for the development of depression. These above data demonstrated that chronic administration of different dermatology drugs contributed into common mental distress. This surprising discovery of chemical stressors stimulating the hippocampal dysfunction, paves the way for exciting areas of study on the cross-talk between the skin and the brain, as well as is suggesting how to develop effective and safe usage of dermatological drugs in daily practice.

## 1. Introduction

Dermatology diseases are a common global clinical problem, which significantly affects the patients’ quality of life and causes enormous financial burden. More than 2,000 different types of dermatology disease entities are prevalent in the world, but the medication security of drugs has not been paid increasing attention. Dermatosis is a chronic disease characterized by intense itching, chloasma and eczematous lesions, etc. Emollient and cream afford some symptomatic and temporary relief, but disease exacerbations often require long-term treatment with topical delivery, the prolonged and larger-body application of which can be associated with dermal atrophy, local swelling and other side effects, such as emesis and diarrhea [1,2]. Experimental researchers also emphasized that dermal absorption of some drugs through topic administration, occupational contact and cosmeceutical exposure can cause a number of local effects, including irritant and allergic contact dermatitis [3,4]. Clinical trials indicate that potential risks of dermatology products constitute a wide spectrum ranging from dermal toxicity to systemic toxicity, such as neurotoxicity [5]. In 2007, there had been cases of adverse events reported for Lindane Shampoo and Lindane Lotion in which a serious outcome (hospitalization, disability or death) has occurred. Lindane neurotoxicity, verified by autopsy was the cause of one infant's death, and was the cause of death reported for an adult in a successful suicide. The Dermatology Life Quality Index (DLQI) is a widely accepted patient-oriented score used in many trials. In trials, the reporting of safety is very little standardized. Neuropsychiatric aspects have also naturally been neglected after dermatology treatment.

Earlier, the skin is considered to be “a mirror of the soul” and “our brain on the outside” [6,7]. Epidermal and dermal cells secrete and respond to classical stress neurotransmitters, neuropeptides, and hormones. Such production is stimulated by ultraviolet radiation (UVR), biological factors (infectious and noninfectious), and other physical and chemical agents [8]. Agents from the outside world passes through the epidermal layers, the first line of immune defense, and then interacts with the neuroendocrine system [7]. The classic neuroendocrine axes such as the cutaneous hypothalamic-pituitary-adrenal axis (HPA) and hypothalamic-thyroid axis also exist in the skin, making the skin be an important peripheral neuro-endocrine-immune organ [8,9]. The progenies of skin structures are stemmed from neurogenesis, although the epidermis and hair follicles are later populated with neural crest-derived melanocyte and merkel cells [9]. It has already been established that the skin is an important peripheral neuro-endocrine-immune organ that is tightly networked to central regulatory systems. All skin structures are innervated by an extensive neural network of somatosensory and antonomic nerve fibers [8]. Cutaneous afferent nerve terminals transduce sensory stimuli in response to temperature, PH, pressure, chemical, and inflammation, with projections to the specific areas of the brain [9]. Environmentally stressed skin can activate both the central and local HPA axis through either sensory nerves or circulating hormonal factors to turn on homeostatic responses counteracting cutaneous and systemic damage [9–12]. Multiple levels of cutaneous systems including pigmentary, immune and endocrine, are present that seperate noxious from benign stimuli. During this processing, these skin systems can also communicate with brain either through chemical messengers entering circulation or through direct neural signals [9,13]. In clinical, psychoderrmatology describes an interaction between dermatology and psychiatry. The incidence of psychiatric disorders among dermatological patients is estimated at about 30 to 60% [14]. Based on the above warning mental health problems in the daily usage of dermatologic drugs should be urgent. Herein, we selected three most common dermatology drugs (HQ, INN, TAC) with transdermal delivery, which mainly regulate cutaneous pigmentary system, sebaceous gland function (peripheral endocrine function), and immune system, respectively, and then analyzed their neurological dysfunction. Transdermal drug delivery are performed to have systemic effects as a results of the distribution of the active compound through systemic circulation, and especially topic applications exhibit local effects as a result of persisting in the skin in order to monitor how alterations of cutaneous stress response systems affect brain functions.

**Fig 1:**
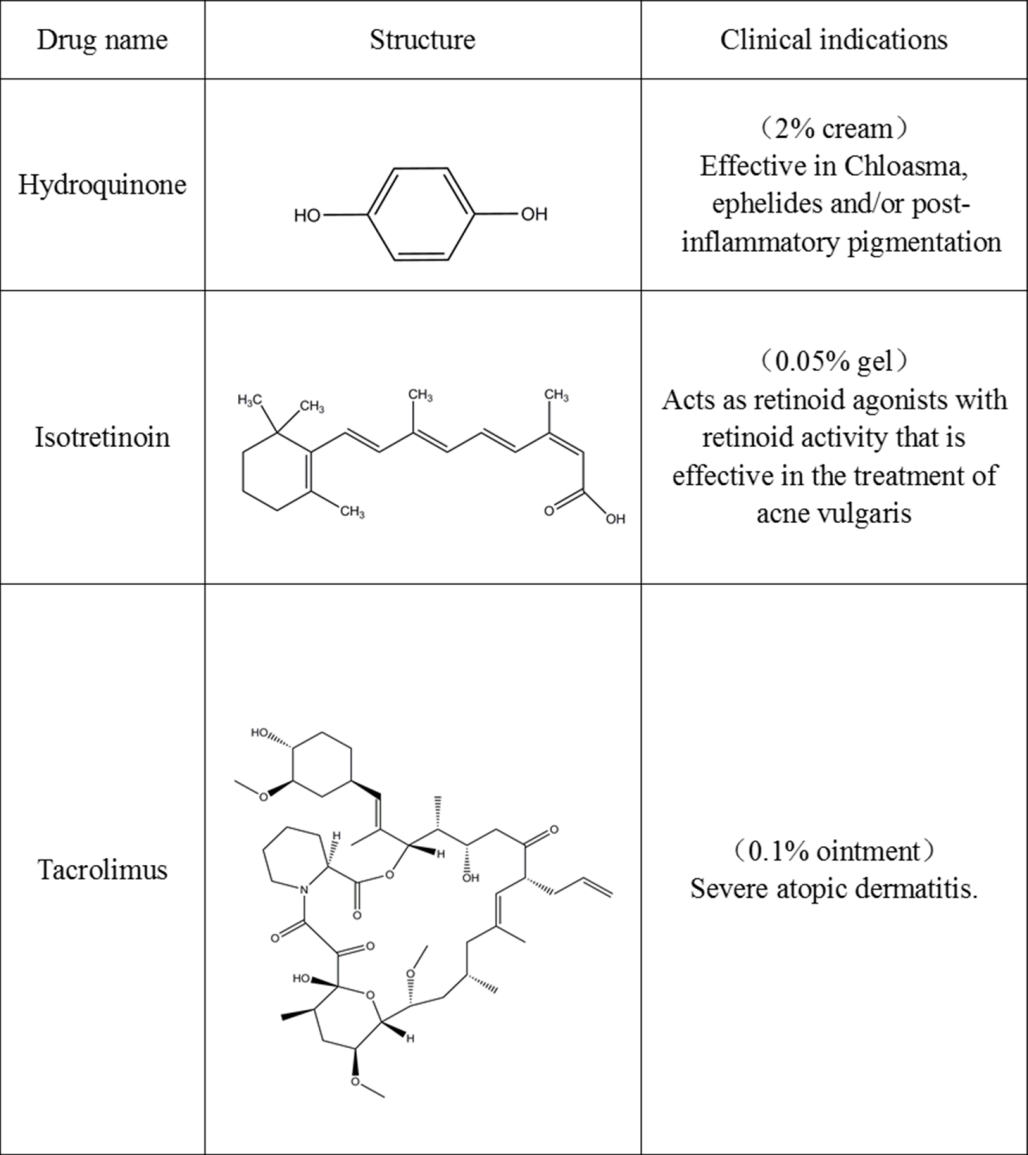
Structure and Indications of drugs used in the experiment.

HQ, a phenolic chemical compound well known as 1, 4-dihydroxybenzene, acts by inhibiting the enzymatic oxidation of typosine and phenoloxidases. It is a strong oxidant that is rapidly converted to the melanocyte toxic substances, p-benzoquinone and hydroxybenzoquinone. These by products cause skin depigmentation [15]. Second, INN (13-cis retinoic acid, Accutane), is approved by the FDA for the therapy of acne. It works for the treatment of acne by inhibiting sebaceous gland, which exhibits an independent peripheral endocrine function and expresses receptors for corticotrophin-releasing hormone (CRH) system [16]. Finally, TAC is a 23-membered macrolide lactone isolated from the bacterium *streptomyles tsukubaensis*. TAC has been mostly used a calcineurin inhibitor immunosuppressant for organ transplantation, applied intravenously and orally. A topic treatment of TAC has also potent immunosuppressive activity [17]. It inhibits calcineurin action, thus preventing T-cell activation and the production of various inflammatory cytokines to the treatment for atopic dermatitis and vitiligo. These above drugs obviously have different structures and different clinical indications (**Fig 1**).

Some studies have indicated that the relationship between skin and brain exists due to more than a fact, that the brain, as the center of psychological function, and the skin, have their same ectodermal origin and are affected by the same neuro-hormones and neurotransmitters [9]. We aim to provide more evidence that how cutaneous systems (pigmentation, endocrine and immune) impact the brain functions in response to external chemical stressors HQ, INN, and TAC.

## 2. Materials & Methods

### 2.1. Animals

Male C57BL/6 mice (5 weeks old, weighing 18~20g, Certification No. scxk (Su) 2012–0004) were obtained from the Laboratory Animal Service Center of Yangzhou University. The animals were housed under controlled temperature (23 ± 1°C) and humidity (50 ± 5%) with a 12–hour light/dark photoperiodic cycle (lights on at 6:00 a.m. and off at 6:00 p.m.). Food and water were provided *ad libitum*. The experimental procedures were conducted in accordance with the guidelines of the “the National Institutes of Health guide for the care and use of Laboratory animals (NIH publications No. 8023, revised 1978)” and have been approved by the Animal Experimentation Ethics Committee of the Chinese Pharmaceutical University.

### 2.2. Experimental Procedure

**Fig 2:**
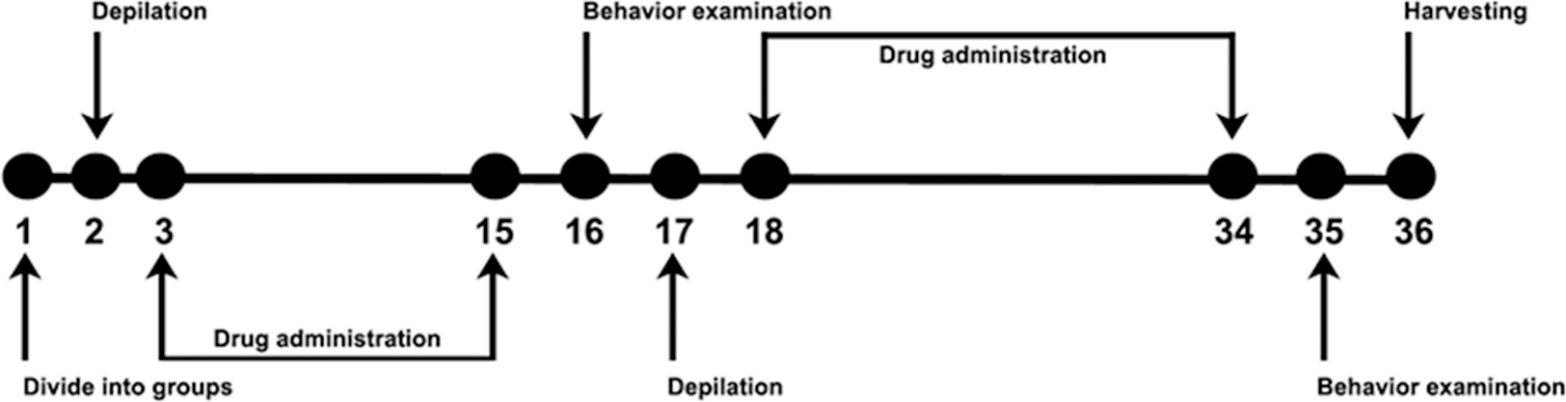
Schematic illustration of the experiment. During the schedule, behavior tests were performed on day 16 and 35 to evaluate the neurotoxic effects of dermatology drugs. Depilation was carried out on day 2 and 17 to induce anagen of hair cycle as described previously [19]. Dermatology drugs were transdermally delivered for total 30 days.

After adaptation to the environment for 3 days, mice were randomly divided into several groups according to the behavioral assessment results. In experiment 1, to determine whether three dermatology drugs could induce common mental status changes, mice were allocated into the following five groups: control group (without hair removal and other treatments), hair removal group, 2% HQ, 0.05% INN and 0.1% TAC group. There were 10 mice in every group. In experiment 2, to enable sensitive monitoring the dose-response characteristics of the neurobehavioral disorders, C57BL/6 mice were divided into six groups: control group, hair removal group, 0% (only treated with base material), 2%, 4% and 6% HQ group. There were also 10 mice in every group. On the second day, wax/rosin mixture (1:1 on weight) was applied to induce a highly synchronized hair growth as described previously [18]. From day 3 to day 15, the mice were treated with dermatology drug for one hair growth cycle. At day 16, the first behavior test was operated. Then mice were secondly performed the procedures of depilation. From day 18 to day 34, drug was continuously administrated. At day 35, the second behavior examination was operated and mice were sacrificed for the next neurochemical analysis. **(Fig 2)**.

### 2.3. Drug and Treatment

Topical HQ was oil-in-water emulsion cream which contains water 61.3%, stearic acid 8.0%, white vaseline 8.0%, glycerol 7.0%, octadecanol 6.0%, propylene glycol 5.0%, azone 2.0%, trolamine 1.6%, sodium dodecylsulfate 1.0%, ethylparaben 0.1%. The concentrations of HQ were 0, 2, 4 and 6% (w/w). Oxidation of HQ to benzoquinone was controlled by the addition of anhydrous sodium sulphite (all reagents were obtained from sinopharm chemical reagent co., Ltd, China). Topical 0.05% INN gel (Stiefel Laboratories Ltd., Ireland) and 0.1% TAC ointment (Astellas Toyama Co., Ltd., Japan) were also used to treat a test patch of skin in mice.

The administered dose was designed according to the clinical dosage. In the first experiment, the dosage of three drugs administrated to mice is 0.01g HQ cream/20g body weight, 0.25g INN gel/20g body weight and 0.01g TAC ointment/20g body weight. They are designed to be approximately 5 times of the clinical dose. In the second experiment, skin bleaching was induced by administration of 0.08g cream/20g body weight. 2% HQ cream was approximately 50 times of the clinical equivalent dose.

### 2.4. Open-Field Test

The locomotor activity was evaluated as described previously [20]. The apparatus was a square, walled arena (50 cm × 50 cm × 22 cm) with white Plexiglas and floor. Each of the mice was placed in the center area of the open-field and analyzed for its motility in a time-period of 5 minutes. During a test period of 5 min, the numbers of crossings (squares crossed with all paws) and rearings (rising on the hind paws) were scored by observers who were blind to the treatment conditions. In this test, the locomotor activity was indicated by the numbers travelled in the apparatus while the vertical activity was assigned by number of rearings. At the end of testing, the number of fecal boli was also measured and the arena was cleaned with a 10% ethanol solution.

### 2.5. Tail Suspension Test

The tail suspension test was carried out as previously described [20,21]. Briefly, an adhesive tape was fixed to the mouse tail (distance from the tip of the tail = 2 cm) and hooked to a horizontal ring stand bar placed 30 cm above the floor. All the animals were suspended for 5 min, and the test sessions were video-taped for scoring. Mice were considered immobile only when they hung passively and completely motionless. The immobility time was recorded by observers blind to the treatment conditions.

### 2.6. Tissue Preparation

On day 36, mice were sacrificed under deep anesthesia. For western blot and LC-MS, brain and hippocampus were removed rapidly without perfusion and stored in the liquid nitrogen until use; for immunofluorescence, immunohistochemistry, HE and TUNEL assay, mice were perfused transcardially with 0.1M PBS (Ph 7.4) followed by a fixative solution containing 4% paraformaldehyde in PBS (Ph 7.4) as previously described [19]. The brains were removed and fixed in the same fixative for additional 6~12 h in 4°C. For LC-MS, the hippocampus was precisely weighed to perform the procedure of the homogenate according to per 50 mg tissue adding 1 ml acetonitrile. Homogenate extraction according to our previous protocol [22] was frozen in -20°C for LC-MS examination.

### 2.7. Hematoxylin-Eosin (HE) Staining

HE staining was performed using an HE staining kit (Solarbio, Beijing, China) according to the manufacturer's instructions.

### 2.8. TUNEL Staining

To determine apoptosis-like cell death, TUNEL assay (Beyotime Institute of Biotechnology, China) was performed 24 h after reperfusion (n = 4). Briefly, sections were dewaxed and rehydrated. After treated with proteinase K for 10 minutes at 37°C, the labelling reaction was performed using a solution containing terminal deoxynucleotidyl transferase, its buffer, and fluorescein dUTP at 37°C for 60 minutes in a humidity chamber. Following incubation, the sections are mounted on coverslips with Anti-fade Fluorescence Mounting Medium.

### 2.9. Immunohistochemistry

The fixed brain tissues were embedded in paraffin and were sectioned into 3-μm slices for immunohistochemical staining for the 5-HT1A receptor using an anti-5-HT1A receptor antibody (Abcam) (with a maximum of 6 sections per animal). Briefly, sections were pre-treated using heat mediated antigen retrieval with sodium citrate buffer for 20 mins. Then sections were blocked in 5% normal goat serum diluted in PBS with 0.25% Tween 20. The rabbit polyclonal antibody against 5-HT1A was used at 1:1000 for 15 mins at room temperature and detected using an HRP conjugated compact polymer system. DAB was used as the chromogen. The sections were examined under a laser scanning confocal microscope (Olympus, FV1000).

### 2.10. Immunofluorescence

This experiment was performed as previously described with some modifications [23]. Sections were dewaxed, rehydrated and immersed in citric acid buffer for antigen retrieval. Then after being washed with 0.01 M PBS, the specimens were treated with PBS containing Tween 20 (PBST) for 15 min at room temperature and then blocked for 1 h in blocking buffer (5% goat serum, 0.1% bovine serum albumin, and 0.1% Triton X-100). Thereafter, the specimens were incubated with each of the primary antibody mixtures (5-HT_1B_ receptor, 1: 100, Santa Cruz Biotechnology Inc., California) at 4°C for 24 h. After being washed with 0.01 M PBS, the specimens were incubated with a secondary antibody solution (FITC conjugated goat anti-rabbit IgG, Cwbiotech, CW0114S, China) and in the dark inside a cassette at 37°C for 2 h. The specimens were then washed with 0.01 M PBS and mounted using 50% glycerol and then were observed and photographed under a fluorescence

### 2.11. Western Blot Analysis

At the end of the experiment, hippocampus was quickly dissected out and then lysed in 400 μL RIPA buffer (50 mM Tris-HCl (pH 7.4), 150 mM NaCl, 1 mM PMSF, 1 mM EDTA, 1% Triton X-100, 0.5% sodium deoxycholate, and 0.1% SDS). Western blot analysis was carried out as previously described [24]. Then, after centrifugation at 12.000 rpm/min for 20 min at 4°C, 20 μg of total protein of each sample was loaded into a 12% SDS-PAGE gel and then transferred to PVDF membranes (Millipore). The membrane was blocked with 5% non-fat dry milk in TBS containing 0.05% Tween-20 (TBS-T) for 1 h and incubated overnight with primary Abs (Bax, Bcl-2, Cleaved caspase-3, Caspase 8 and Caspase 9: Cell Signaling Technology Inc., Massachusetts; 5-HT1A receptor: Abcam Inc., UK; 5-HT1B receptor: Santa Cruz Biotechnology Inc., California), mouse polyclonal antibodies against β-actin (1:1000, Sigma–Aldrich, Missouri). After reaction with the second antibody, proteins were visualized by an enhanced chemiluminescence detection system. Densitometric analysis was again carried out by using the Quantity One (Bio-Rad) to scan the signals. Western blot assay results were representative of at least 3 independent experiments.

### 2.12. LC-MS Examination

Chromatographic separations were performed by Finnigan Surveyor LC-TSQ Quantum Ultra AM LC-MS system (Thermo Finnigan, USA) equipped with Xcalibur1.1 workstation, a quaternary pump, an online degasser and a thermostatically controlled column compartment. The mobile phase was composed of (A) water (0.2% formic acid, 0.1% ammonium acetate, v/v) and (B) acetonitrile. The gradient elution was 2–8% B at 0–6 minutes, 8–70% B at 6–8 minutes, 2% B at 8.01 minutes, 2% B at 8.01–12 minutes. Chromatographic separation was carried out at a Hanbon Lichrospher C18 column (4.6mm × 25cm, 5 μm) with a solvent flow rate of 1.0 ml/minute at a temperature of 30°C. The sample injection volume was set at 10 μl.

### 2.13. Statistical Analysis

All data were expressed as means ± SEM. Statistical analysis was performed with One-Way Analysis of Variance followed by Tukey’s post hoc test for multiple comparisons tests. Normality and homoscedasticity of the data were verified before any statistical analysis using levene’s test. Significant differences were accepted when *P* < 0.05.

## 3. Results

### 3.1. Effect of 0.05% INN, 0.1% TAC or 2% HQ Treatments on Behavioral Alterations in C57BL/6 Mice

**Fig 3:**
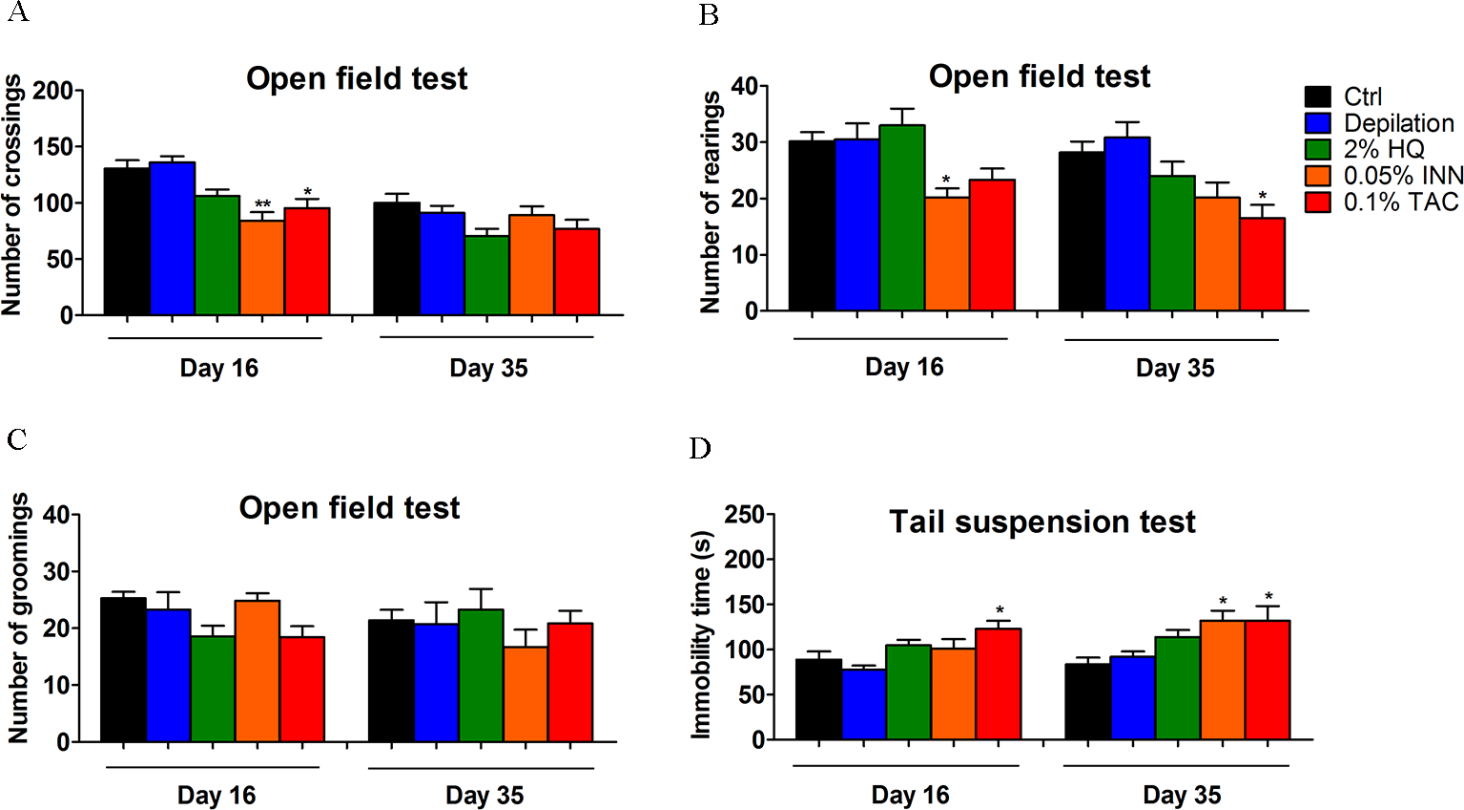
Effect of 2% HQ, 0.05% INN and 0.1% TAC on behavioral activities in C57BL/6 mice. In open field test, a significant decrease was observed in the crossing (A) and rearing numbers (B) of INN and TAC group during 5minutes, whereas no change in the number of grooming episodes (C). In the tail suspension test, INN and TAC significantly elevated the immobility time (*P* < 0.05) (D). Values were expressed as mean ± SEM, n = 6. Data were analyzed by One-Way Analysis of Variance (ANOVA). *P < 0.05, **P < 0.01 *vs*. control group.

In this experiment, to determine whether treatment with three dermatology drugs could result in similar alterations in the neurobehavioral parameters, HQ, INN and TAC (0.01g HQ cream/20g body weight, 0.25g INN gel/20g body weight and 0.01g TAC ointment/20g body weight) were administrated to mice, which were designed to be approximately 5 times of the clinical dose. Repeat administration of HQ, INN and TAC for 35 days did not lead to the development of overt toxicity in treated mice. All animals survived to day 16 and day 35, and there were no difference in the body weight over the treatment period. When percutaneously treated with INN and TAC for 16 days, the crossing and rearing activity were significantly reduced in the open field test **(Fig 3A and 3B**). However, there is no change in the number of grooming episodes (**Fig 3C**). After prolonged exposure with INN or TAC for 35 days, approximatedly 66.7% and 100% of mice presented a lower locomotion **(Fig 3A)**. Control animals all show the ability to habituate to a novel home environment. Here, habituation is defined as a decrease in the three behavior variables crossing, rearing and grooming in response to the diminished novelty of the test chamber. Furthermore, on day 35 INN and TAC significantly elevated the immobility time in tail suspension test (*P* < 0.05) **(Fig 3D)**, with **~**33.3% and 50% mice becoming despair. 2% HQ has no significant behavioral alterations **(Fig 3**).

### 3.2. Effect of 0.05% INN, 0.1% TAC or 2% HQ Treatments on Morphology and Apoptosis in Hippocampus

Numerous studies about the main mechanisms of depression-like behavior have paid special attention to the hippocampus, a brain region critically involved in the regulation of mood, learning/memory and cognitive function [25,26]. Thus, hippocampal structure and neurochemical were investigated following INN and TAC-induced behavioral impairment.

**Fig 4:**
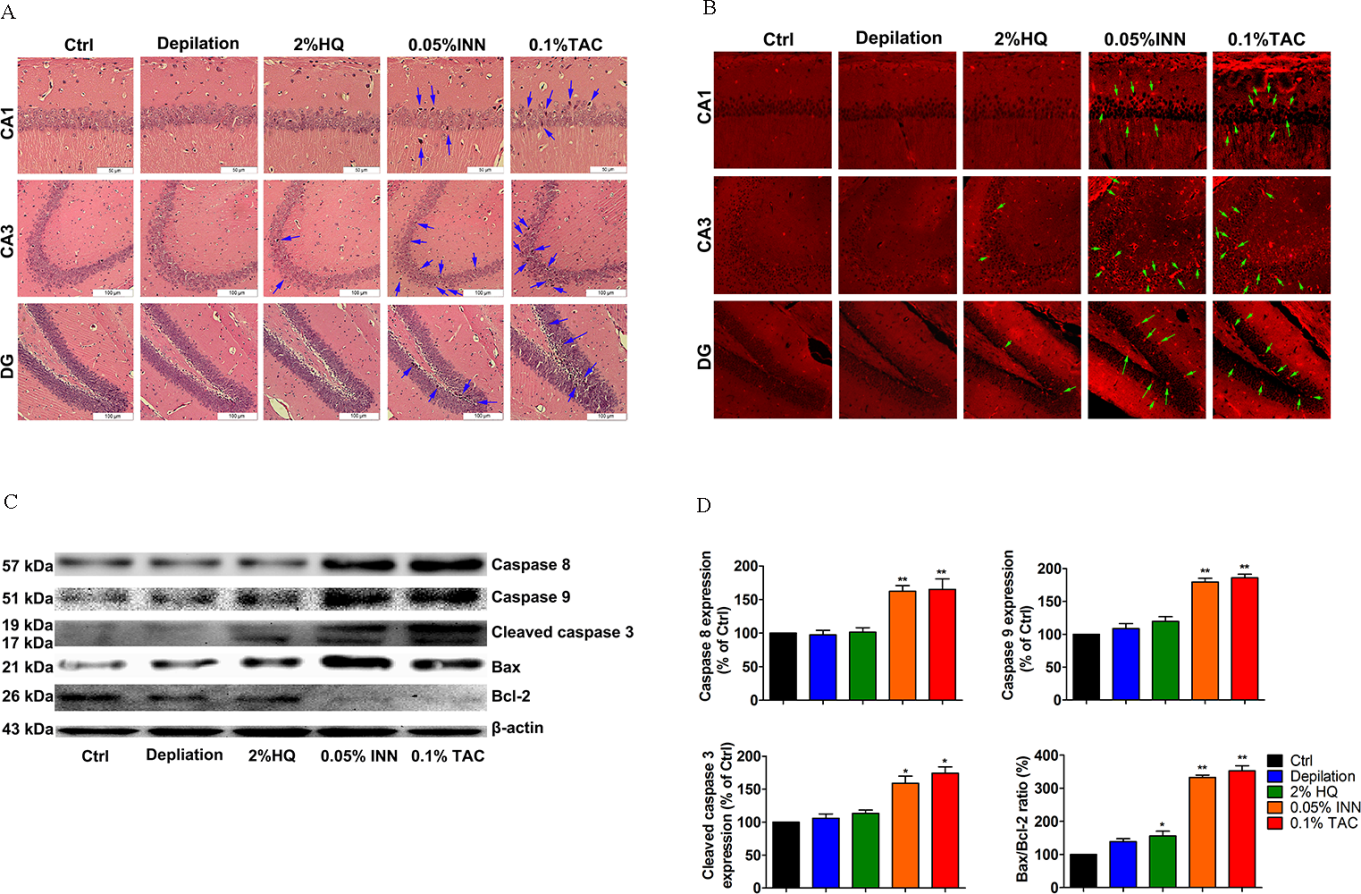
Effect of 2% HQ, 0.05% INN and 0.1% TAC on neuronal damage and apoptosis of the hippocampus in C57BL/6 mice. (A) HE staining showed a higher percentage of cell loss in CA1 region of INN and TAC treated mice. Scale bar = 100μm. (B) Representative photomicrographs showed a striking increase in TUNEL-positive apoptotic cells (indicated by arrow) of hippocampus (CA1, CA3 and DG). Scale bar = 100μm. (C) Hippocampal cell was lysed and total cell extracts were analyzed by western blot. Relative changes in protein expression of pro- or anti-apoptotic proteins (caspase 8, 9, cleaved caspase 3, Bax and Bcl-2) in the hippocampus. (D) Quantitative results for protein expression and bax/bcl-2 ratio following HQ, INN and TAC treatment. Densitometric scanning of band intensities obtained from three separate experiments was used to quantify protein expression. Data were analyzed by One-Way Analysis of Variance (ANOVA). *P < 0.05, **P < 0.01 *vs*. control group.

Neuron injury in hippocampus was analyzed by hematoxylin-eosin staining while survival was measured by TUNEL staining and western blot. INN or TAC treatments caused karyopyknosis in the CA1 field of hippocampus. Accordingly, neuronal density of hippocampus was littler changed **(Fig 4A**). As shown in **Fig 4B**, TUNEL-positive apoptotic neurons in the hippocampus were significantly increased compared to the control. Subsequently, cleaved caspase 3, caspase 8, 9 protein expressions and bax/bcl-2 ratio were augmented in the hippocampus of the INN and TAC group (**Fig 4C and 4D**). Caspase 8, 9 and cleaved caspase 3 expression in the mice administed 0.05% INN or 0.1% TAC, was increased to ~65% and 70% **(Fig 4D**), respectively. In the hippocampus of mice treated with 0.1% TAC, Bax/BCL-2 ratio was 279% of untreated control animals **(Fig 4D**). In conclusion, in contrast to 2% HQ, INN and TAC treatments induced the obvious structural injury and neuronal apoptosis in the hippocampus of the C57BL/6 mice.

### 3.3. Effect of 0.05% INN, 0.1% TAC or 2% HQ Treatments on 5-HT Level and 5-HT_1A/1B_ Expression in Hippocampus

**Fig 5:**
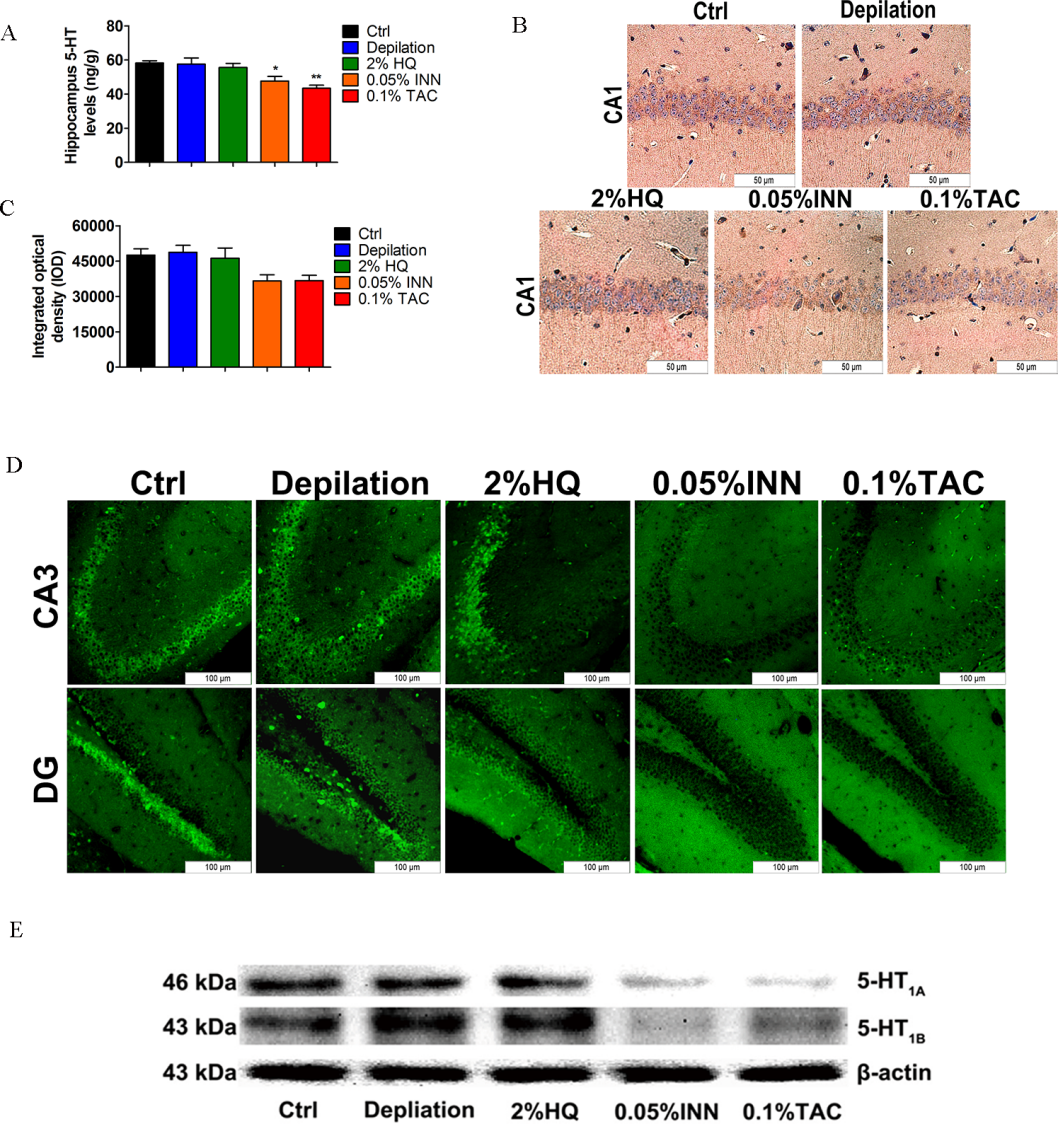
Effect of 2% HQ, 0.05% INN and 0.1% TAC on hippocampal 5-HT level and 5-HT_1A /1B_ receptor expression. (A) Hippocampal 5-HT levels after 2% HQ, 0.05% INN and 0.1% TAC treatments. Each bar represents mean ± SEM (n = 6). (B) The 5-HT_1A_ receptor in the CA1 region was detected by immunohistochemistry. Scale bar = 50μm. (C) IOD values of the expression of 5-HT_1A_ receptor in CA1 region. Data are expressed as mean ± SEM (n = 4). (D) The 5-HT_1B_ receptor expression in the CA3 region and DG was assessed by immunofluorescence. Scale bar = 100μm. (E) The expression of 5-HT_1A_ and 5-HT_1B_ receptors was decreased in INN and TAC-treated mice. Data were analyzed by One-Way Analysis of Variance (ANOVA). *P < 0.05, **P < 0.01 *vs*. control group.

5-HT is well known to be a critical neurotransmitter, which can affect every aspect of brain activity and take part in regulating adult hippocampal neurogenesis [27]. Among at least 21 different 5-HT receptor subtypes, 5-HT_1A/1B_ plays a central role in the regulation of serotonergic neurotransmission and has been implicated in the etiology and treatment of psychiatric disorders (stress response, anxiety and major depression, behavior) [28]. And 5-HT_1B_ binding sites are rich in CA3 region and dentate gyrus of the hippocampus [29]. Principal neurons in hippocampal CA1 region expressed 5-HT_1A_ receptor mRNA, and CA3 pyramidal neurons displayed low 5-HT_1A_ receptor expression [30]. Thus, using LC-MS we found that INN and TAC could suppress the synthesis of 5-HT to ~10% and 15% (**Fig 5A**). Protein expression of 5-HT_1A/1B_ was evaluated in the mice hippocampus (CA1, CA3 and DG region) by immunohistochemistry, immunofluorescence and western blot (**Fig 5B, 5C, 5D and 5E**). Administration of 0.05% INN and 0.1% TAC caused a decrease in the number of cells immunopositive for 5-HT_1A_ within the CA1 region of the hippocampus (**Fig 5B and 5C).** The 5-HT_1B_ receptor in the CA3 and DG region was detected by immunofluorescence. All animals from INN and TAC groups showed similar immunopositive signals, with displayed obvious lower expression (**Fig 5D**). In the end, downregulation of the hippocampal 5-HT_1A/1B_ receptor by INN and TAC exposure was validated using western blot (**Fig 5E**). HQ was found to be no significant effect on hippocampus 5-HT-5-HT_1A/1B_ system, which was consistent with no neurobehavioral distress. These molecular evidences indicate that INN or TAC produce behavioral disorders, probably mediated by 5-HT-5-HT_1A/1B_ dysfunction.

### 3.4. Effect of Gradient Concentrations of HQ Treatment on Alteration of Neurobehavioral Activities

**Fig 6:**
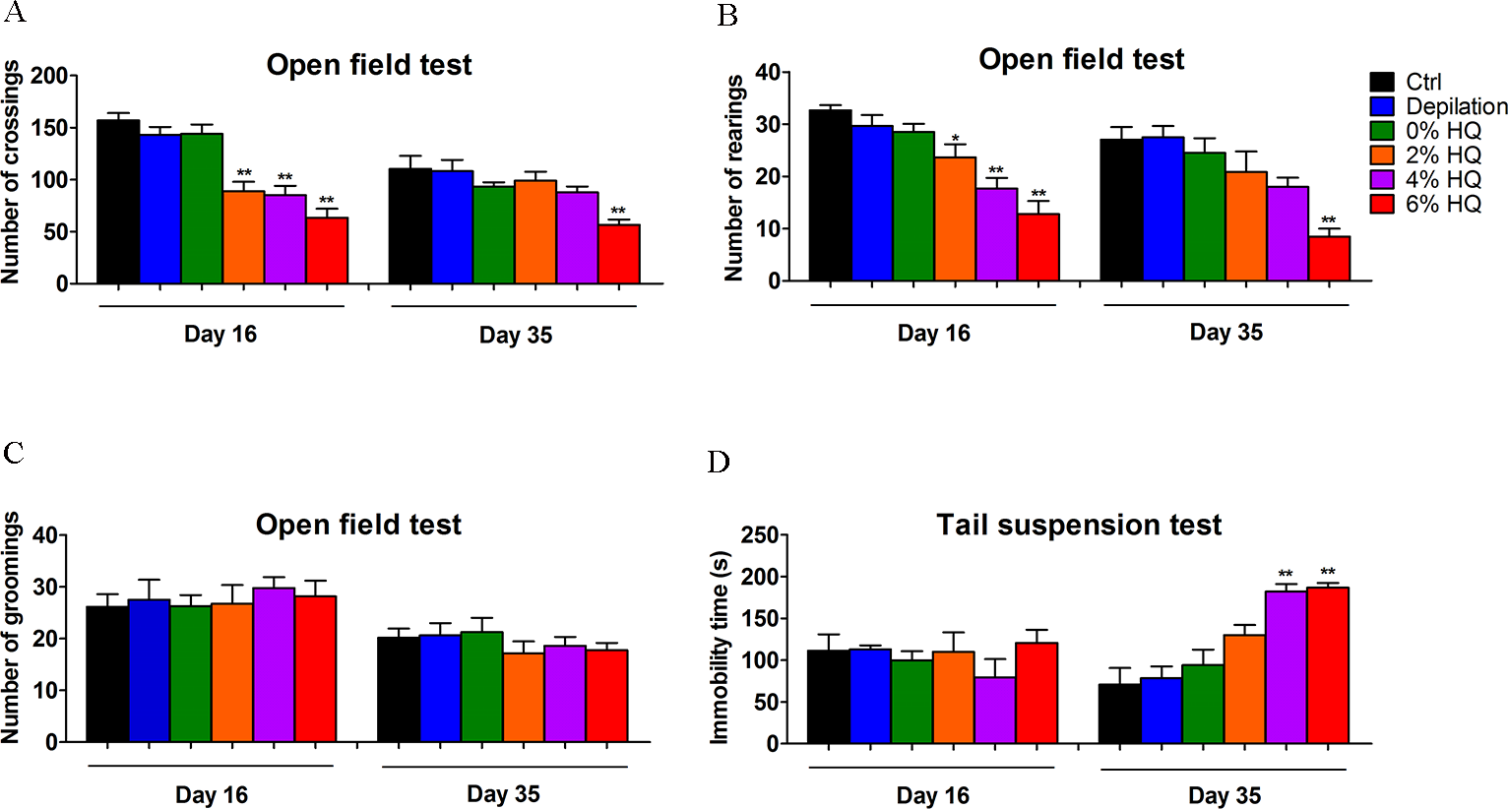
Effect of 2%, 4%, and 6% HQ cream on behavioral activities. In open field test, a significant decrease was observed in the crossing (A) and rearing numbers (B) during 5minutes. No significant differences were shown in grooming numbers (C). In the tail suspension test, 4% and 6% HQ cream-treated groups displayed longer immobility time (D) on day 35. Values were expressed as mean ± SEM, n = 6. Data were analyzed by One-Way Analysis of Variance (ANOVA). *P < 0.05, **P < 0.01 *vs*. control group.

In clinical 2% HQ is used to treat Chloasma, ephelides and/or post-inflammatory pigmentation. For moderate-to-severe hyperpigmentation, we are often required long-term and larger body application. Thus, under this condition, to further provide more evidences on psychotic symptoms of HQ dose-dependent response, mice received gradient concentrations of hydroquinone (2%, 4%, 6%, 2% (5000 mg/kg) equal to 50 times clinical dosage). At day 16 and 35, the spontaneous activity and behavioral desperation were assessed by open-field test (OFT) and tail suspension test (TST) respectively. When compared with control group, HQ did not cause significant changes in the grooming frequency (**Fig 6C**). However, at day 35, 37.5% of 2% HQ mice, 50% of 4% HQ mice and 100% of 6% HQ mice displayed a distinct decrease in locomotion activity, respectively (**Fig 6A**). Meanwhile, 33.3%, 80% and 100% mice from three HQ treatments all showed a decreased tend to rearing activity (**Fig 6B**). All data indicate that the effects of HQ on habituation ability had a dose-dependent response. Further, prolonged treatment for 35 days also showed a dose-dependent increase of the immobility time in TST, with 16.7%, 83.3% and 100% HQ mice becoming desperation (**Fig 6D**). These results reflected that HQ-treated mice were accompanied by the state of behavioral despair.

### 3.5. Effect of Gradient Concentrations of HQ Treatment on Cytoarchitecture and Survival in the Hippocampus

**Fig 7:**
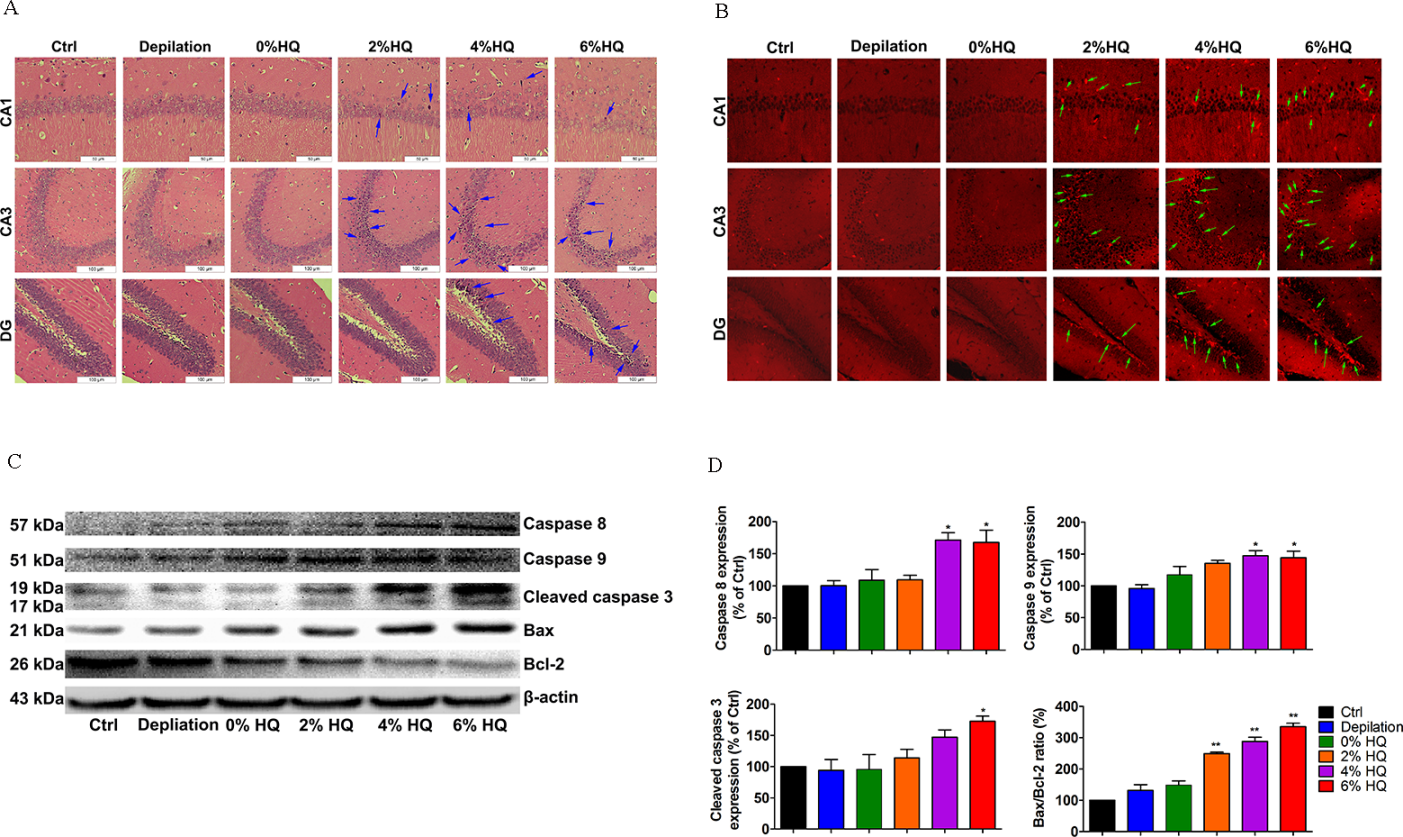
Effect of 2%, 4%, and 6% HQ cream on neuronal damage and apoptosis of the hippocampus. (A) HE staining showed a much higher percentage of hippocampal cell loss in CA1 region of HQ cream-treated mice. Scale bar = 100μm. (B) Representative photomicrographs revealed a striking increase in TUNEL-positive apoptotic cells of hippocampus (indicated by arrow). Scale bar = 100μm. (C) Hippocampal cell was lysed and total cell extracts were analyzed by western blot. Quantitation of western blot analysis for Caspase 8, Caspase 9, Cleaved caspase 3, Bax and Bcl2. (D) Quantitative results for pro- or anti-apoptotic protein expression and bax/bcl-2 ratio following HQ-treated concentration at 2%, 4%, and 6%. Densitometric scanning of band intensities obtained from three separate experiments was used to quantify proteins expression. Data were analyzed by One-Way Analysis of Variance (ANOVA). *P < 0.05, **P < 0.01 *vs*. control group.

Numbers of pyramidal cells in CA1 region were strikingly decreased in HQ-treated groups (4%, 6%). Nonetheless, numbers of granule cells in CA3 region and dentate gyrus showed no significant change (**Fig 7A**). As shown in **Fig 7B**, in contrast to the control group, the numbers of TUNEL-positive cells in the HQ-treated groups were significantly increased. Neuron survival in hippocampus was further evaluated in HQ-treated mice by western blot. The up-regulation of apoptosis-related proteins (cleaved caspase 3, caspase 8, 9) expression also occurred in dose-dependent manner (**Fig 7C**). Besides, HQ (2%, 4%, 6%) exposure increased hippocampal bax/bcl-2 ratio to 158%, 196% and 248%, respectively (**Fig 7D**). Thus, these results imply that HQ-treated mice rendered a variety of neurobehavior, probably through the impairment of hippocampal structure and survival.

### 3.6. Effect of Gradient Concentrations of HQ Treatment on 5-HT Level and 5-HT_1A/1B_ Receptors Expression in Hippocampus

**Fig 8:**
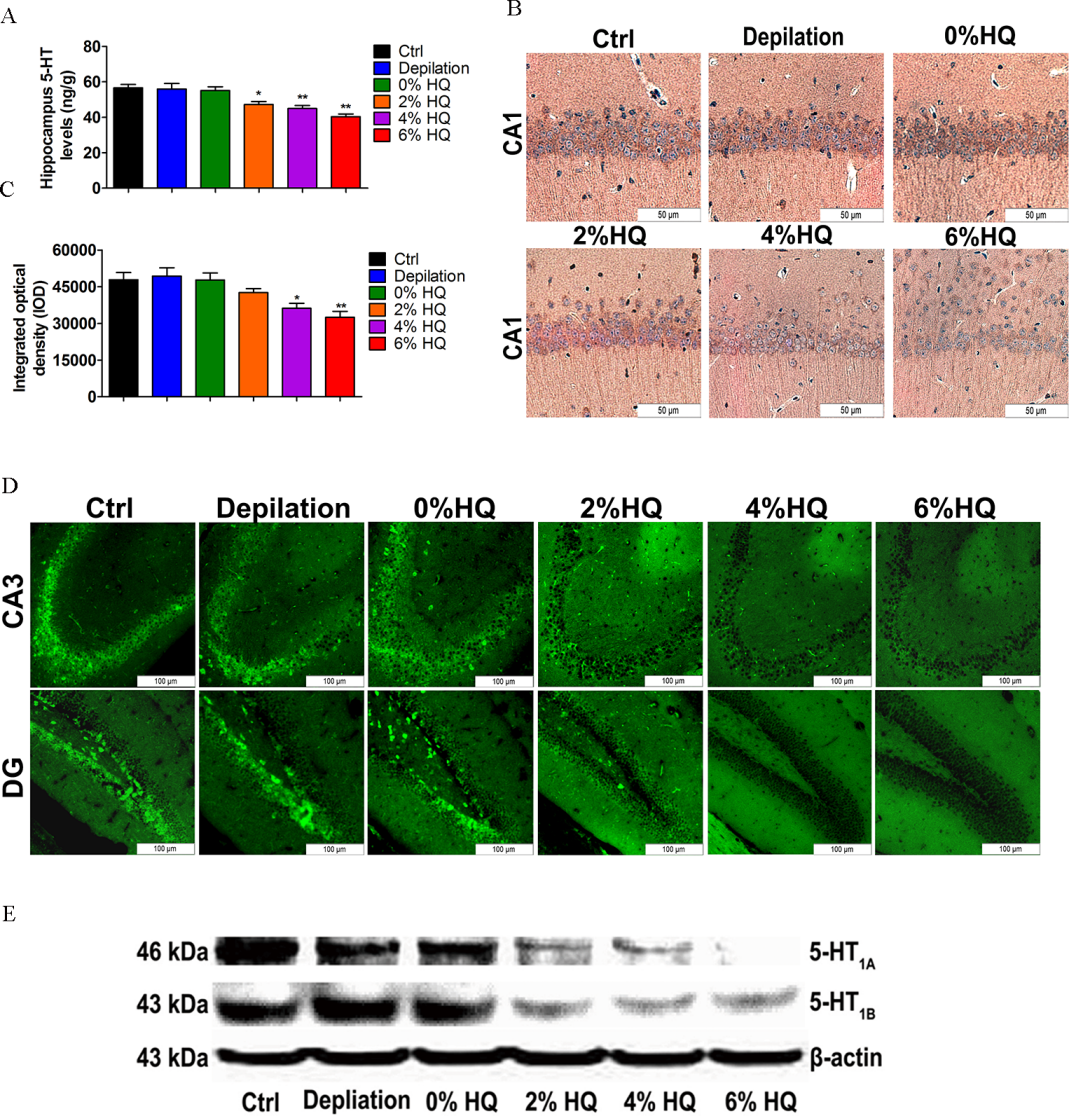
Effect of 2%, 4%, and 6% HQ cream on hippocampal 5-HT level and 5-HT_1A /1B_ receptor expression. (A) HQ decreases hippocampus 5-HT level in a dose-dependent manner. Each bar represents mean ± SEM (n = 6). (B) The 5-HT_1A_ receptor in the CA1 region was detected by immunohistochemistry. Scale bar = 50μm. (C) Integrated optical density (IOD) values of the expression of 5-HT_1A_ receptor in CA1 region. Each bar represents mean ± SEM (n = 4). (D) The 5-HT_1B_ receptor in the CA3 region and dentate gyrus was detected by immunofluorescence. Scale bar = 100μm. (E) The expression of 5-HT_1A_ and 5-HT_1B_ receptors in the hippocampus was down-regulated in a dose-dependent manner. Data were analyzed by One-Way ANOVA. *P < 0.05, **P < 0.01 *vs*. control group.

After C57BL/6 mice received HQ, the detection of 5-HT level and 5-HT_1A/1B_ expression in the hippocampus was also performed. We found that the level of 5-HT in hippocampus displayed a dose-dependent decrease by LC-MS assay (**Fig 8A**). Then, the expression of 5-HT_1A/1B_ receptors was collectively validated by immunohistochemistry, immunofluorescence and western blot assay. The results showed that HQ also had an obvious inhibitory expression of 5-HT_1A/1B_ receptors in a concentration-dependent manner (**Fig 8B, 8C, 8D and 8E**). These results indicate that HQ-induced neurotoxic effects are related to the dose-response characteristics of hippocampal 5-HT-5-HT_1A/1B_ expression.

## 4. Discussion

It is generally recognized that dermatology drugs always remain on the surface of the body, and local toxicity was the primary safety concern with percutaneous delivery. During the experiment period, no obvious signs of toxic symptoms were observed, such as body weight, immune system, atrophy and swelling, etc. The measured body weights were noted during the time of drug exposure. There was no significant difference between the body weights (**S1 Fig**) and thymus and spleen index (**S2 Fig**) of the control mice compared to the mice exposed to HQ, INN and TAC. Also, HQ-treated mice were characterized by depigmentation, no atrophy or no swelling in dorsal coat (**S3 Fig**). Similarly, arbutin, production of HQ, has been reported to be related to development of pigmentary changes [31]. It is evident that local safety was seen in the dosal skin of three drugs treatment. In contrast, the prolonged and larger-dosage application contributed to severe psychiatric disorders (behavior dysfunction, hippocampus abnormalities). This neurotoxicity displayed a dose-dependent response. Compared with the control group, the crossing and rearing frequency in open field test reduced more conspicuously in INN and TAC-treated mice (**Fig 3A and 3B**). Elevated immobility time in tail suspension test was also induced (**Fig 3D**) whereas the behavioral activities in 2% HQ was unchanged. In the acute studies reported, the effects following HQ administration to rodents are fairly characteristic. Tremors were observed at all dose levels (a range of 100–500 mg/kg, ip) in both the range-finding study and the full acute toxicity study [32,33]. Stimulatory effects of HQ on the CNS have not been reported following dermal exposure. For example, dermal doses of 3840 mg/kg (rats) and 4800 mg/kg (mice) for 14 days did not produce adverse CNS effects in NTP studies [33]. Then, to further sensitive monitor neurotoxic effects of HQ treatment, mice were administrated with three gradient concentrations of HQ (2%, 5000mg/kg; 4%, 10^4^mg/kg; 6%, 1.5×10^4^mg/kg). Doses of 4% and 6% HQ have significantly altered scores in open field test (**Fig 6A and 6B**) and tail suspension test (**Fig 6D**). These behavioral abnormalities were found in 100% of 6% HQ mice through OFT and TST examination, thereby reminding us a potential underlying psychiatric disorder after longer exposure to larger doses of HQ. These results are consistent with some previous findings. Consumption of large acute doses of HQ-containing mixtures, accidentally or with suicidal intent, has been reported to produce signs of acute CNS disorders [34]. INN is a confirmed human teratogen and its use is contraindicated during pregnancy due to the serious risk of characteristic patterns of anomalies in CNS [35,36]. This agent is a synthetic derivative of vitamin A and more resistant to catabolism. It is similar to the parent compound in being fat-soluble. As expected, topic INN into the systemic circulation was high, with systemic exposure to INN being approximately 22 times higher than that in the epidermis [37]. It can across the blood-brain barrier easily and act in the brain tissue [38]. Since it crosses the blood-brain barrier, INN affects the expression of a wide spectrum of genes in the limbic structures, thus changing the function of the dopaminergic, serotonergic, noradrenergic neurons involved in the modulation of mood [38]. Our results also suggest that INN can induce depressive behavior **(Fig 3)**, which is associated with the prevetion of hippocampal 5-HT-5-HT1A/1B system and induces apoptosis **(Figs 4 and 5)**. Nevertheless, this depressive behavior did not follow immediately after treatment but mostly 35 days after INN treatment. This phenomenon is line with that within clinical cases. Review of these cases indicates that depression and suicide do not occur immediately after treatment but commonly 1~2 months after commencement, sometimes with a longer delay [39]. This suggests that the mechanism may not be via immediate influence of INN on a crucial neurotransmitter (5-HT) **(Fig 5A)** or other signal pathway(s) but may be through a secondary system.

Another very interesting example is the impact of TAC on the brain. Being a highly lipophilic drug, diffusion into the systemic circulation was minimal, with systemic exposure to TAC being 750 and 1800 times lower than that observed in the skin at 24 h after first and last application, respectively [40,41]. Thus TAC is primarily partitioned in the skin, with minimal systemic absorption after topical application. Accordingly, TAC exerts immunosuppressive effects on the immuncompetent cells in the epidermis and dermis [17,42]. An overwhelming proportion of studies are focused on emergence of neurotoxicity in a stable immunosuppressed renal transplant patient [43,44]. However, there are no clinical neurotoxicity concerns associated with the ointment. In our studies, we investigated the effect of topic TAC (cutaneous immunosuppressive) on the brain functions. The above data had shown that topic TAC could induce behavioral distress at the early period (Day 16), accompanied by the down-regulation of hippocampal 5-HT-5-HT1A/1B systems and the induction of neuron death **(Fig 4)**. There needs to be a plausible mechanism of this action. TAC is as a macrolide antibiotic produced by *streptomyles tsukubaensis* with strong T-specific, immunosuppressant activity. Here, the expression of several inflammatory T-cell cytokines is inhibited. Indeed, topic TAC downregulates skin proinflammatory cytokines, including IL-2, IL-3, IL-4, IL-5, IFN-c, TNF-α [45]. The latter can enter the systemic circulation. Presumably, it is also possible that TAC affects brain either via neutrally transmitted signals or via secondary effects such as cellular components of the peripheral immune system (lymphocytes, mast cells, eosinophils, and dendritic cells (DCs)). The effect of skin local immunity system on depressive status suggests that the direct functional connection between the skin and the brain can be through skin immune response. Topic HQ is the standard prescription treatment of facial dyspigmentation [15]. Consumption of larger acute doses of HQ, accidentally or with suicidal intent, has been reported to produce signs of acute CNS distances in clinical cases and rodent studies [33]. Here dermal doses of 2% (C57BL/6 mice) for 16 days did cause adverse behavioral effects **(Fig 6)**. Similarly, several adverse events were observed at greater dose levels **(Fig 6)**. HQ has a cytotoxic effect on hippocampus by apoptosis induction and 5-HT-5-HT1A/1B system inhibition, and the pro-apoptotic signaling is involved in caspase 3/9 pathway **(Fig 7)**. A possible explanation for HQ neurotoxity is due to local depigmentation or a rapid formed metabolite. Depigmentary signals (melanocyte apoptosis) generated from the skin in response to HQ may induce a secondary cascade of pro-inflammatory or activate some receptors located on cutaneous sensory nerve endings to alert the brain of changes, thereby to induce behavioral disturbances. The amounts of HQ that have been measured in rat brain are relatively low after single and repeated applications [46] indicating that HQ can not accumulate in the brain. Our results also provide some evidence for a dose response effect for HQ and psychiatric side effects, where higher doses are associated with more side effects. We concluded that the “psychological changes in brain may be dose related”.

The serotonergic system has complex interactions with many neurotransmitter systems, and plays a key modulatory role in CNS. It has been reported that hippocampus is a crucial regional distribution of 5-HT-moduline and 5-HT receptors binding sites. Diversity of serotonergic receptor subtype and region-specific innervations is involved in mediating various physiological, emotional, and higher-order cognitive processes [47]. A growing body of evidence suggests the special interaction between the serotonin system (including 5-HT and its receptors, for instance, 5-HT_1A/1B_ receptors) and skin functions [7,20,48–50]. Serotonin may be associated with drug-induced adverse effects in the central nervous system (CNS), which could help define the relationship between the skin and neuropsychiatric conditions. Accordingly, our data showed that in contrast to 2% HQ, INN and TAC produced progressive inhibition of the synthesis of 5-HT **(Fig 5A)**, and the expression of 5-HT_1A/1B_ protein in the hippocampus **(Fig 5B, 5C, 5D and 5E)**. The level of 5-HT and the expression of 5-HT_1A/1B_ receptors were both gradually decreased in gradient concentrations of HQ-treated group **(Fig 8A, 8B, 8C, 8D and 8E)**. More importantly, by increasing caspase 3, 8, 9 and Bax/Bcl-2 ratio **(Fig 4C and 4D and Fig 7C and 7D)**, three drugs impairs neuronal growth which is necessary to the cognition and behavior processes. Consequently, above neurochemical deficit (5-HT-5-HT1A/1B system, survival-related proteins) may be closely related to neurobehavioral dysfunction.

**Fig 9:**
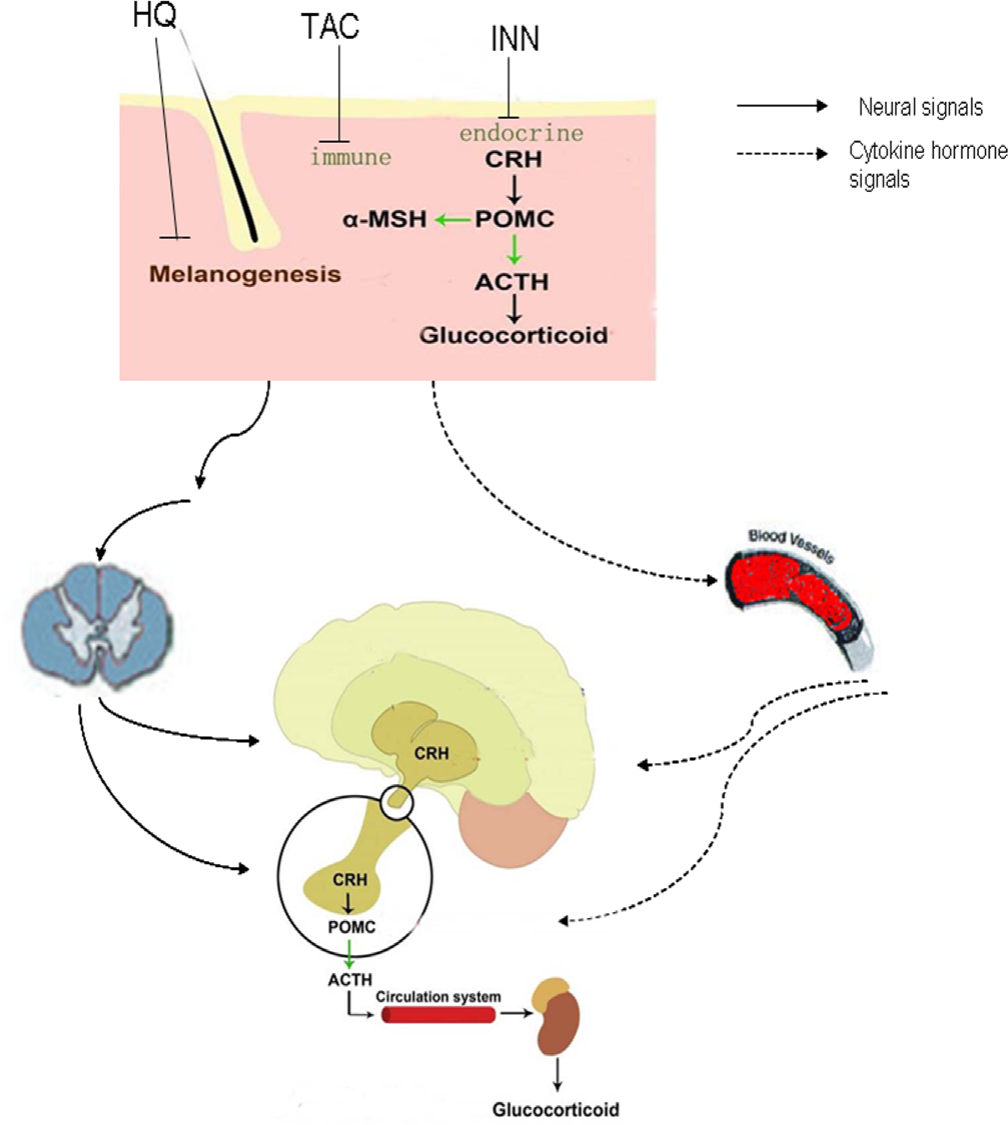
Skin-brain cross-talk upon exposure to HQ, INN and TAC. Skin stress system in response to HQ, INN and TAC may activate the central HPA with direct homeostatic, phenotypic consequences, thereby contributing to brain dysfunction. Modification from Fig 1 published in “Sensing the environment: Regulation of local and global homeostasis by the skin neuroendocrine system”[8].

An interesting aspect of the current study is the observed common behavioral disorders elicited by mice treated with HQ, INN or TAC. These are three different types of dermatology drugs, differing in their structure, therapeutic use and their pharmacological mechanisms and yet the behavioral neurotoxic characteristics i.e., the modified spontaneous behavior to a novel home environment and the depressive behavior, were similar and durable through transdermal delivery. Described as the body's largest organ, the skin is strategically located at the interface with the external environment where it has evolved to detect, integrate and respond to a diverse range of stressors [51]. Here, we can consider three chemical agents as the external stressors. It is early well recognized that skin stress response system can activate the central HPA axis with direct homeostatic, metabolic and phenotypic consequences [8,9,52]. Certainly it is easy to understand that a common contributing mechanism (central HPA axis) is involved to these neurotoxic effects. Local signals in response to HQ, INN and TAC are either delivered by ascending nerve routes to the brain or by circulation to hypothalamus to activate CRH production **(Fig 9)**. This indicates that a common contributing mechanism is implicated with the functional connection between skin systems and brain functions.

## 5. Conclusion

The present study demonstrated that the neurotoxic effects, in altered spontaneous behavior and desperation, were commonly shown in C57BL/6 mice treated with HQ, INN or TAC. Our data also suggest that changes in 5-HT-5-HT_1A/1B_ or neuron survival-related proteins may contribute to disorders in the developing brain. Reversely, these neurochemical changes may be a contributing factor to the neurobehavioral dysfunction. Although some receptors and apoptosis proteins stand out in the involvement of the neurotoxic influences, it is unlikely that a single 5-HT_1A/1B_ receptor or protein has the sole responsibility in this complex cascade of underlying mechanisms involved with the neurobehavioral function. There should be other important mechanisms such as central HPA axis that need to be further explored in the future. Also, assessing the neurotoxic impacts of different dermatology drugs, it makes us get a better understanding of the functional connections between the skin and the brain. This would further be beneficial to the finding of potential prediction or prevention for the clinical therapy. Once the disorder has been diagnosed, management requires a dual approach, addressing both dermatologic and psychologic aspects. The cooperation of the dermatologist and the pshchiatrist in order to increase the life quality of the patients is of utmost importance.

## Supporting Information

S1 Fig Comparison of body weights. Mice were treated with 0%, 2%, 4%, 6% HQ cream (A) or 2% HQ, 0.05% INN, 0.1% TAC (B). The body weight was recorded throughout the study. No significantly statistical difference was observed *vs*. control group. Statistical differences were calculated with a one-way ANOVA test.
(TIF)

S2 Fig Comparison of immune tissue index. Mice were treated with 2%, 4%, 6% HQ cream (A) or 2% HQ, 0.05% INN, 0.1% TAC (B). The thymus and spleen index was measured after the treatments. Results were presented as mean ± SEM. No statistically significant difference was observed *vs*. control group.
(TIF)

S3 Fig Macroscopic observations of the skin pigmentation. Mice were treated with 2%, 4%, 6% HQ cream (A) or 2% HQ, 0.05% INN, 0.1% TAC (B). The color of corresponding area in the dorsal skin was shown. The skin pigmentary function of HQ cream-treated groups was reduced. HQ cream-treated groups were whiter than hair removal group in a dose-dependent manner.
(TIF)

## Abbreviations

5-HT: 5-hydroxytryptamine
5-HT1A/1B: 5-hydroxytryptamine 1A receptor and 1B receptor
HQ: Hydroquinone
INN: Isotretinoin
TAC: Tacrolimus
TUNEL: transferase-mediated deoxyuridine triphosphate-biotin nick end labeling
FITC: fluorescein isothiocyanate
Bcl-2: B-cell lymphoma-2
Bax: Bcl2-Associated X
OFT: open-field test
TST: tail suspension test
CNS: central nervous system
