# Supplementary figures and images for: Developmental Neurotoxic Effects of Percutaneous Drug Delivery: Behavior and Neurochemical Studies in C57BL/6 Mice
Source: PLoS One. 2016 Sep 8;11(9):e0162570. doi: 10.1371/journal.pone.0162570 (PMC5015905; doi:10.1371/journal.pone.0162570)

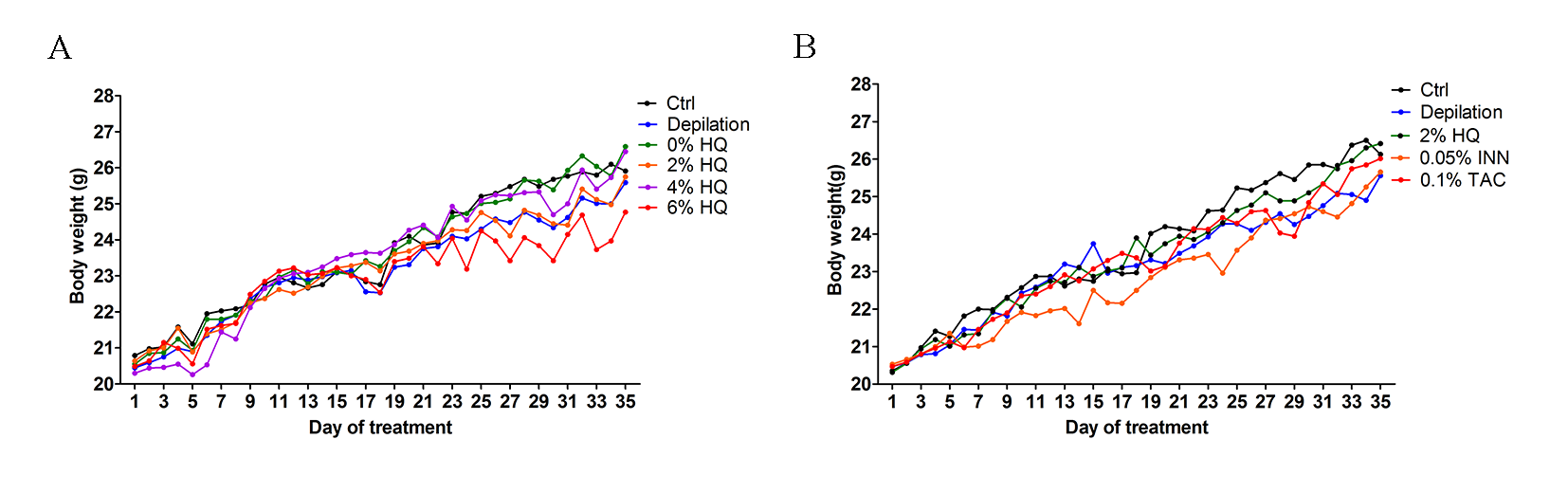

Supplement: S1 Fig — Mice were treated with 0%, 2%, 4%, 6% HQ cream (A) or 2% HQ, 0.05% INN, 0.1% TAC (B). The body weight was recorded throughout the study. No significantly statistical difference was observed vs. control group. Statistical differences were calculated with a one-way ANOVA test. (TIF) [file pone.0162570.s001.tif]

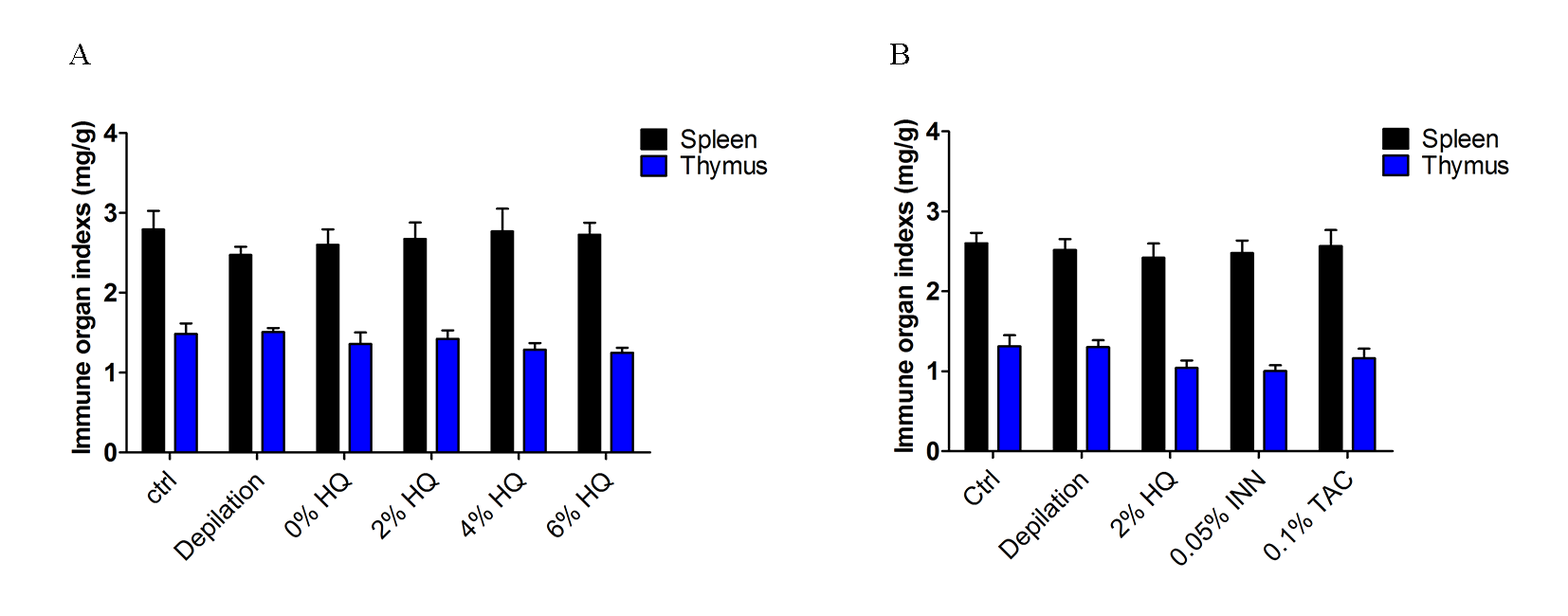

Supplement: S2 Fig — Mice were treated with 2%, 4%, 6% HQ cream (A) or 2% HQ, 0.05% INN, 0.1% TAC (B). The thymus and spleen index was measured after the treatments. Results were presented as mean ± SEM. No statistically significant difference was observed vs. control group. (TIF) [file pone.0162570.s002.tif]

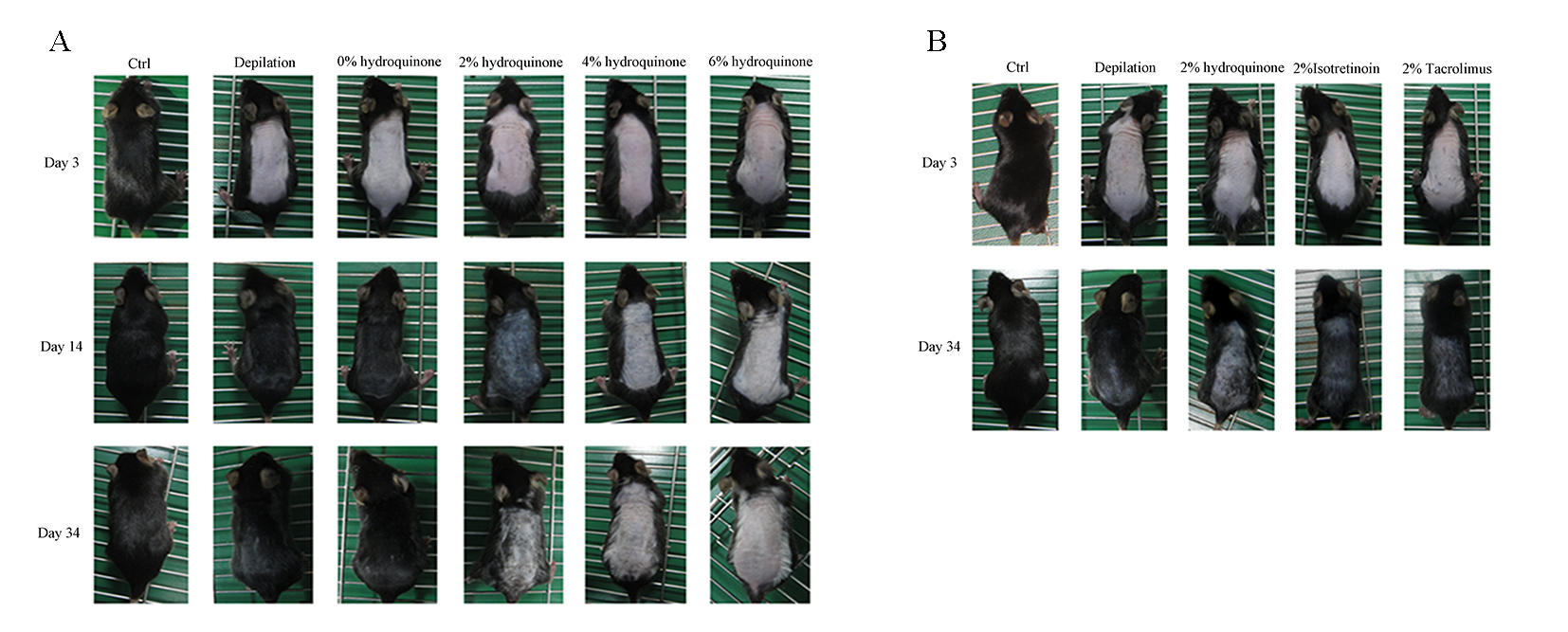

Supplement: S3 Fig — Mice were treated with 2%, 4%, 6% HQ cream (A) or 2% HQ, 0.05% INN, 0.1% TAC (B). The color of corresponding area in the dorsal skin was shown. The skin pigmentary function of HQ cream-treated groups was reduced. HQ cream-treated groups were whiter than hair removal group in a dose-dependent manner. (TIF) [file pone.0162570.s003.tif]
